# Massive Ovarian Mucinous Cystadenoma Complicating Term Birth

**DOI:** 10.7759/cureus.64054

**Published:** 2024-07-08

**Authors:** Sudwita Sinha, Simran Simran, Mukta Agarwal, Upasna Sinha, Tarun Kumar

**Affiliations:** 1 Obstetrics and Gynaecology, All India Institute of Medical Sciences, Patna, Patna, IND; 2 Radiology, All India Institute of Medical Sciences, Patna, Patna, IND; 3 Pathology/Lab Medicine, All India Institute of Medical Sciences, Patna, Patna, IND

**Keywords:** term birth, mucinous cyst adenoma, gravid period, pregnancy, ovarian cyst

## Abstract

Usually, symptomatic ovarian cysts in pregnancy require surgical removal in the second trimester. However, occasionally, large ovarian cysts may be encountered in the third trimester, which might hinder normal vaginal delivery. Herein, we present one such case to highlight the challenges of managing a large ovarian cyst in a full-term pregnancy.

## Introduction

The widespread use of ultrasound in prenatal care has resulted in a rise in the identification of ovarian cysts during pregnancy [1]. Pregnancy-induced physiological and anatomical alterations might cause discomfort, leading to the potential oversight of pathological conditions such as ovarian tumours in the absence of regular scans [1]. When caring for a woman, it is crucial to evaluate her medical condition, symptoms, gestational age, type of tumour, size, origin, and how it may impact her pregnancy [1]. Expectant management can be done in asymptomatic cases with close monitoring of the mother, and surgical management can be planned at term [1].

## Case presentation

A 34-year-old, G3P2L2, with two previous vaginal deliveries, was referred to us by a private practitioner in the periphery at 37 weeks, six days of gestation, with undue enlargement of the abdomen for one month, which was associated with pain for the past three to four days. She had sought prenatal care three times earlier and had not experienced any discomforting symptoms till then. An ultrasound scan conducted at 14 weeks detected a significant mass-multiloculated cyst with low-level internal echoes without any features of malignancy in the abdominal and pelvic area, measuring 15 cm × 12 cm, located in the left adnexa. By 23 weeks, the mass had grown to 20.8 cm × 13.8 cm, and in the near term, it reached a size of 27 cm × 17 cm, multilocular with low-level internal echoes with no evidence of thick septations, vascularity, or solid areas. There was no significant change in the ultrasound appearance of the mass over time during pregnancy. The patient was getting her antenatal checkup from a private practitioner, who had referred her to a tertiary centre for evaluation of the growing mass during pregnancy. However, the patient did not consider it necessary as she was not having distressing symptoms due to the growing mass per se. Term foetal USG showed a single live intrauterine foetus with a placenta in the fundo-anterior position, an amniotic fluid index of 11 cm, an estimated foetus weight of 2.9 kg, and normal Doppler parameters. Upon evaluation, the patient's vital signs were stable. An abdominal examination showed a tense and unduly enlarged abdomen. The uterus was occupying the right lumbar, right iliac, and some parts of the right hypochondriac region. The foetal heart rate was normal. Additionally, the middle and left quadrants of the abdomen were occupied by a mass of cystic consistency. The mass was exerting pressure on the uterus, causing it to deviate towards the right side. The uterus could not be centralised, and the foetal head was located in the right iliac fossa region. The foetal head was not engaged.

**Figure 1:**
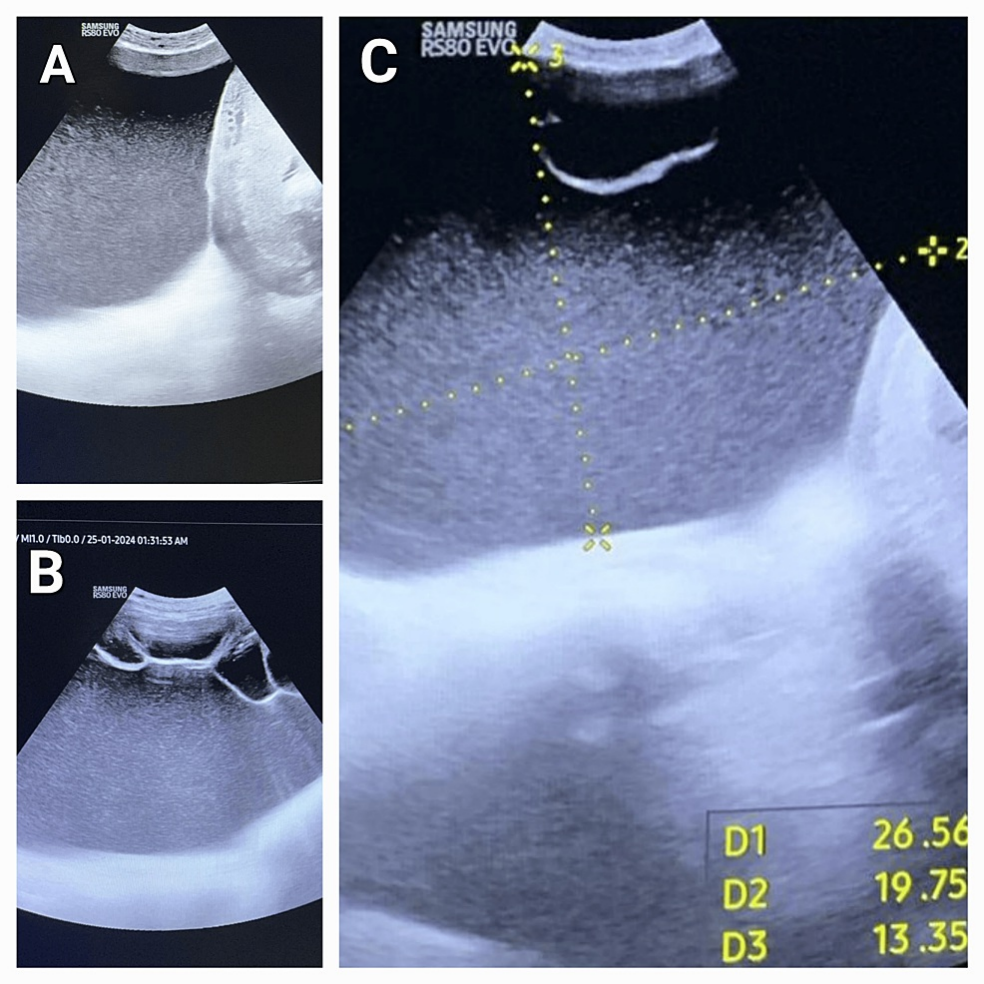
Ultrasonographic image of the ovarian cyst. (A) Ovarian cyst abutting the uterus with the placenta; (B) large multilocular cyst with homogenous low-level echoes in the left adnexal region extending into the abdomen; (C) dimensions of the cysts: 26.56 cm × 19.75 cm × 13.35 cm.

**Figure 2:**
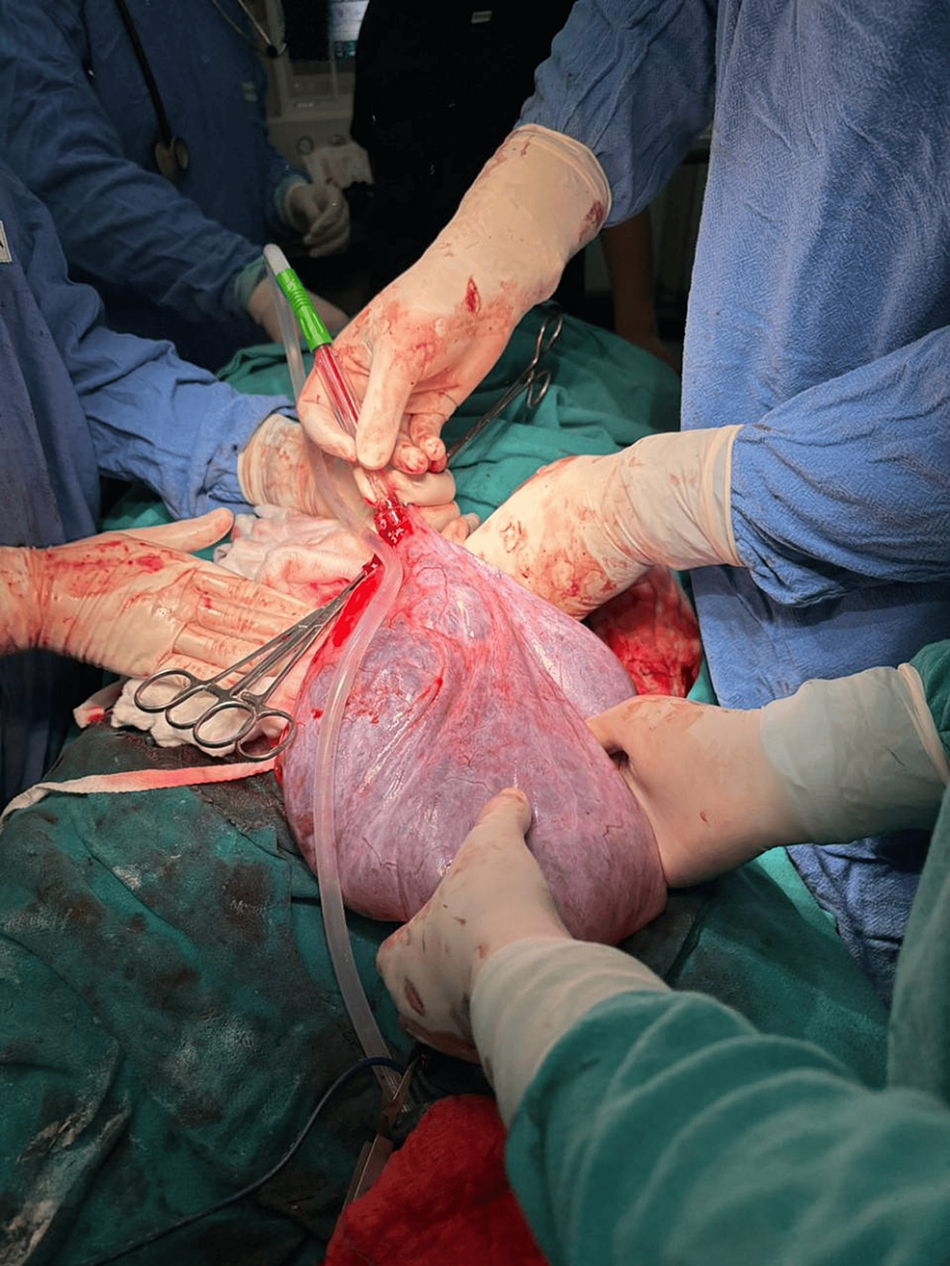
Intraoperative image of the cyst being delivered outside the abdominal cavity after decompression.

**Figure 3:**
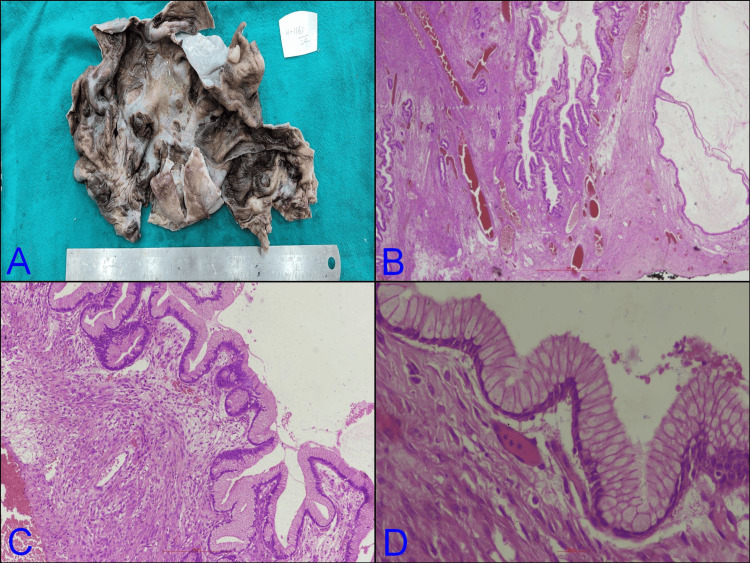
Histopathological images of the ovarian cyst. (A) Grossly, a cut-open specimen of the ovary shows multiple cystic areas; (B) ovarian parenchyma shows multiple cysts and glands lined by simple, non-stratified mucinous epithelium (H&E; 2×); (C) the simple non-stratified mucinous epithelium resembles gastric foveolar type epithelium (H&E; 4×); (D) nuclei are small and basally located without cytological atypia. No invasion has been identified (H&E; 10×).

On vaginal examination, the cervix deviated to the left side, admitting one finger, uneffaced, and the station was high up. The patient underwent routine blood investigations, which showed results within the normal range. Although an MRI (magnetic resonance imaging) was recommended, the patient declined owing to financial limitations. After the initial assessment of the patient, it was evident that normal delivery would not be possible as the uterus was not at all in alignment with the pelvic axis due to the deviation of the uterus towards the extreme right part of the maternal abdomen by the large ovarian mass. On discussing this situation and possible management options (waiting for spontaneous onset and progress of labour versus surgical management) and its outcome with the patient and her attendants, they opted for surgical management. Informed written consent was obtained. Under combined general and epidural anaesthesia, the abdomen was accessed by making a suprapubic transverse incision. A midline longitudinal incision was avoided as there were no radiological features suggestive of malignancy pre-operatively. Moreover, the mass was predominantly cystic in nature, which can be easily decompressed even through a transverse incision and then delivered in toto, taking care to avoid spillage. A live male infant was delivered via a lower segment caesarean section, weighing 2.820 kg, with an APGAR score of 9 at both one and five minutes after birth. There was a large ovarian tumour measuring 27 cm × 20 cm on the left side that extended up to the xiphisternum. The cyst was mobilised, brought to the incision site, and then decompressed by aspirating over 8 L of mucinous fluid with a suction cannula through a stab incision over the most prominent part of the cyst. Great caution was taken to prevent any spillage of the cystic content into the abdominal cavity. After decompression, the cyst was delivered outside the abdominal cavity. A left-sided salpingo-oophorectomy was carried out. The cyst was observed to have lobulated morphology and a smooth surface upon gross inspection. On the cut section, mucinous fluid came out, and there was no solid component. A prophylactic appendectomy was performed as the cyst was of the mucinous type. A mild postpartum haemorrhage with an estimated blood loss of about 800 ml occurred, which was managed medically. After achieving haemostasis and counting mops and instruments, the abdomen was closed in layers. Post-operatively, the patient recovered uneventfully. Bowel sounds were appreciated after four hours of surgery, after which she was allowed oral sips and clear liquids. She passed flatus after eight hours of surgery, following which a soft diet and gradually a normal diet were allowed. She ambulated within 12 hours of surgery, after which Foley’s catheter was removed. She was discharged from the hospital on the third post-operative day in stable condition, along with her baby. The final histopathological examination confirmed the diagnosis of mucinous cystadenoma with focal epithelial proliferation and no features of dysplasia or malignancy. Sections from the appendix showed features of chronic appendicitis with no features of dysplasia or malignancy. On follow-up after six weeks, the patient was in good health with no complaints. Figure 1 shows an ultrasonographic image of a large multilocular cyst with low-level internal echoes in the left adnexal region extending in the abdomen of dimensions 26 cm × 19 cm × 13 cm, abutting the uterus with the placenta. Figure 2 is an intra-operative gross image of the cyst being decompressed. The cut section of the ovary showing multiple cystic areas is shown in Figure 3A, whereas Figure 3B-3D shows histopathological images depicting ovarian parenchyma, a simple non-stratified mucinous epithelium, without cytological atypia or invasion, respectively.

## Discussion

Due to the regular use of ultrasonography in pregnancy, most ovarian masses are incidentally identified and do not exhibit any symptoms. During sonography, if cysts larger than 3 cm are detected, they frequently exhibit self-limiting growth after a period of 10 weeks [1]. Lesions that have complex sonological features (thicker fluid or blood, septations, or solid areas) and persist for more than 12 weeks are referred to as neoplasms, which are often non-cancerous [2-3]. The primary symptom of an ovarian tumour is discomfort, which can vary in intensity from mild to severe in cases of rupture or torsion, requiring immediate surgical intervention [4]. Ultrasound is considered the most reliable method for diagnosing ovarian masses, with a sensitivity of 96.8% and a specificity of 77% [1]. If an MRI is accessible, it may be advised in circumstances where there is ambiguity [3,5]. MRI's capability to visualise the posterior and lateral regions of the pelvis, which may be obscured by foetal bones, confers it an edge over sonography. The procedure is considered safe because of its 100% accuracy rate and the absence of hazardous ionising radiation [6]. The role of tumour markers in pregnancy is not well understood since, when they are studied separately and without the use of imaging techniques, all three indicators - CA125, CEA, and alpha-fetoprotein - are usually elevated during pregnancy and vary over each trimester, making it challenging to draw conclusions [7]. For this reason, we did not recommend tumour markers in our patient either.

Surgery is the primary method of management. The primary complaint, gestational age, and size of the tumour are the most influential factors determining the need for surgical removal.

It is important to always consider the occurrence of preterm labour and intrauterine growth restriction (IUGR) throughout the third trimester, as well as the potential for spontaneous abortion in the first trimester [8]. However, our patient was undergoing antenatal check-ups elsewhere throughout her pregnancy and presented to us only after reaching full term. She did not give a history of any symptoms of preterm labour, nor was there any feature of IUGR. The second trimester is the optimal time for performing surgery to remove ovarian tumours due to the lower level of risk compared to the first or third trimester. However, this was not done for our patient. The probable reason could be that the patient did not have any significant symptoms due to the cyst per se, and her consulting physicians did not feel a need to counsel or discuss this management option with the patient.

Considering the advantages of a shorter hospital stay and decreased blood loss, it may be worth considering laparoscopic treatment for the cyst. However, it is important to note that this approach requires expertise and may not be suitable for women with large tumours or in advanced stages of pregnancy, as was the situation in the above-presented case [9]. It is advisable to reserve surgery for situations of emergency or when symptoms first appear and to use careful and meticulous care. In our case, we opted for surgical management as normal delivery was not feasible and cyst removal could be done together with a lower-segment caesarean section in the same sitting.

Several case reports have indicated that the best approach for large ovarian cysts that develop in the third trimester is to carefully monitor the situation and surgically remove the cyst during a planned caesarean section [10,11]. This was what we also did in our case.

Qublan et al. described a multiloculated right ovarian cystadenoma measuring 33 cm × 24 cm × 20 cm and weighing 6300 g in a pregnant woman at 38 weeks of pregnancy [10]. The condition was associated with IUGR and an abnormal foetal presentation. The woman had a birth weight of 2.5 kg and was born in the breech position. In contrast to this finding, our patient did not have either malpresentation or IUGR. But the size of the cyst was also smaller, which could explain the absence of IUGR in our case.

On reviewing the literature, we could find very few cases of mucinous cystadenoma of the ovary with pregnancy, and among those reported, in the majority of cases, surgical management had been done before the third trimester. Ovarian tumours complicating pregnancy are a rare occurrence. It is usually masked due to pregnancy, unless it is something as large as a mucinous cystadenoma. Hence, keeping in mind the rare conditions helps in tackling such patients, especially in the near term.

## Conclusions

Mucinous cystadenoma presenting at full term is not commonly encountered by an obstetrician. However, if they do encounter it, they should have a clear knowledge and understanding of the possible ramifications of this substantial tumour on pregnancy, the associated complexities, and the necessity for its expeditious extraction. If the patient does not show any symptoms, it may be considered to closely monitor the situation without taking immediate action or having a planned surgery performed at term. However, growing masses and masses changing in appearance during pregnancy may be characteristics that warrant immediate surgery even in pregnancy.
